# Construction and characterization of a novel glucose dehydrogenase-leucine dehydrogenase fusion enzyme for the biosynthesis of l-*tert*-leucine

**DOI:** 10.1186/s12934-020-01501-2

**Published:** 2021-01-06

**Authors:** Langxing Liao, Yonghui Zhang, Yali Wang, Yousi Fu, Aihui Zhang, Ruodian Qiu, Shuhao Yang, Baishan Fang

**Affiliations:** 1 Department of Chemical and Biochemical Engineering, College of Chemistry and Chemical Engineering, Xiamen University, Xiamen, 361005 People’s Republic of China; 2 College of Food and Biological Engineering, Jimei University, Xiamen, People’s Republic of China; 3 The Key Lab for Synthetic Biotechnology of Xiamen City, Xiamen University, Xiamen, Fujian People’s Republic of China

**Keywords:** l-*tert*-leucine, Glucose dehydrogenase, Leucine dehydrogenase, Fusion enzyme, Whole cells

## Abstract

**Background:**

Biosynthesis of l-*tert*-leucine (l-tle), a significant pharmaceutical intermediate, by a cofactor regeneration system friendly and efficiently is a worthful goal all the time. The cofactor regeneration system of leucine dehydrogenase (LeuDH) and glucose dehydrogenase (GDH) has showed great coupling catalytic efficiency in the synthesis of l-tle, however the multi-enzyme complex of GDH and LeuDH has never been constructed successfully.

**Results:**

In this work, a novel fusion enzyme (GDH–R3–LeuDH) for the efficient biosynthesis of l-tle was constructed by the fusion of LeuDH and GDH mediated with a rigid peptide linker. Compared with the free enzymes, both the environmental tolerance and thermal stability of GDH–R3–LeuDH had a great improved since the fusion structure. The fusion structure also accelerated the cofactor regeneration rate and maintained the enzyme activity, so the productivity and yield of l-tle by GDH–R3–LeuDH was all enhanced by twofold. Finally, the space–time yield of l-tle catalyzing by GDH–R3–LeuDH whole cells could achieve 2136 g/L/day in a 200 mL scale system under the optimal catalysis conditions (pH 9.0, 30 *°*C, 0.4 mM of NAD^+^ and 500 mM of a substrate including trimethylpyruvic acid and glucose).

**Conclusions:**

It is the first report about the fusion of GDH and LeuDH as the multi-enzyme complex to synthesize l-tle and reach the highest space–time yield up to now. These results demonstrated the great potential of the GDH–R3–LeuDH fusion enzyme for the efficient biosynthesis of l-tle.

## Background

Due to the tremendous application in marketed drugs, chemical and enzymatic methods of producing l-*tert*-leucine (l-tle), which is widely used as a chiral building block in the synthesis of anti-tumor and anti-virus drugs [1, 2], such as atazanavir, telaprevir and boceprevir, have been developed rapidly. However, the disadvantages were significant and pervasive existed in most chiral l-tert chemical catalysis producing process, including energy-consuming, environmentally-unfriendly, and low conversion and enantioselectivity [3, 4]. The greener biocatalysts, such as leucine dehydrogenase [5], branched chain aminotransferase [6], amidases [7], and proteases [8], penicillinyl enzymes [9], lipases [10], have been developed and applied in l-tle enzymatically producing process over the past few decades. Among those biocatalysts, leucine dehydrogenase (LeuDH, EC 1.4.1.9) exhibited an outstanding conversion efficiency and enantioselectivity which became the main method of l-tle synthesis in the market [11].

In the process of LeuDH production of l-tle, NADH as a cofactor is indispensable and expensive, so an efficient cofactor regeneration system was considered as an ideal solution with high catalytic and economic benefits [11]. In previous research, a cofactor regeneration system was built for the regeneration of expensive cofactor NADH [5, 12, 13], in which amination of trimethylpyruvic acid (TMA) was reductive by LeuDH coupling with Formate dehydrogenase (FDH, EC 1.2.1.2) or Glucose dehydrogenase (GDH, EC 1.1.1.47) to generate NADH. For example, Degussa AG (now Evonik AG) reported a LeuDH and FDH coupling system to perform repeated batch reactions with an enzyme membrane reactor on a ton scale and the space–time yield of l-tle achieved 638 g/L/day [14]. LeuDH and GDH were co-expressed in *E. coli* to constructed whole cells biocatalyst for l-tle production, which obtain space–time yield of 945 g/L/day produce in condition of 1.3 h and 0.5 M substrate concentration [13]. However, the poor structural stability and the high cost of purification and immobilization of free enzyme system limit the industrial application.

Inspired by sophisticated protein complexes in nature [15, 16], artificial multi-enzyme systems have been reported to improve structural stability, promote the cofactor regeneration efficiency and reduce the cost of free enzyme system [17, 18]. LeuDH and FDH with PDZ (PSD95/Dlg1/Zo-1) domain and the corresponding ligand of the metazoan (PDZlig) were fused and assembled as multiple supramolecules to improve the NAD(H) recycling efficiency and structural stability [19]. A series of engineering bifunctional enzyme complex fusing LeuDH and FDH with different peptide linkers were built [20], which could accelerate the transfer of cofactors to increase catalytic rate and stabilize enzyme structure. The multi-enzyme complexes of FDH and LeuDH has been extensively studied, however the low enzyme activity of FDH in the coupling system limited the overall catalytic efficiency [21]. To the best of our knowledge, the GDH may exhibit great potential in the cofactor regeneration of NADH [22], while has not been reported for the construction with LeuDH to form the multi-enzyme complexes in the biosynthesis of l-tle.

**Scheme 1:**
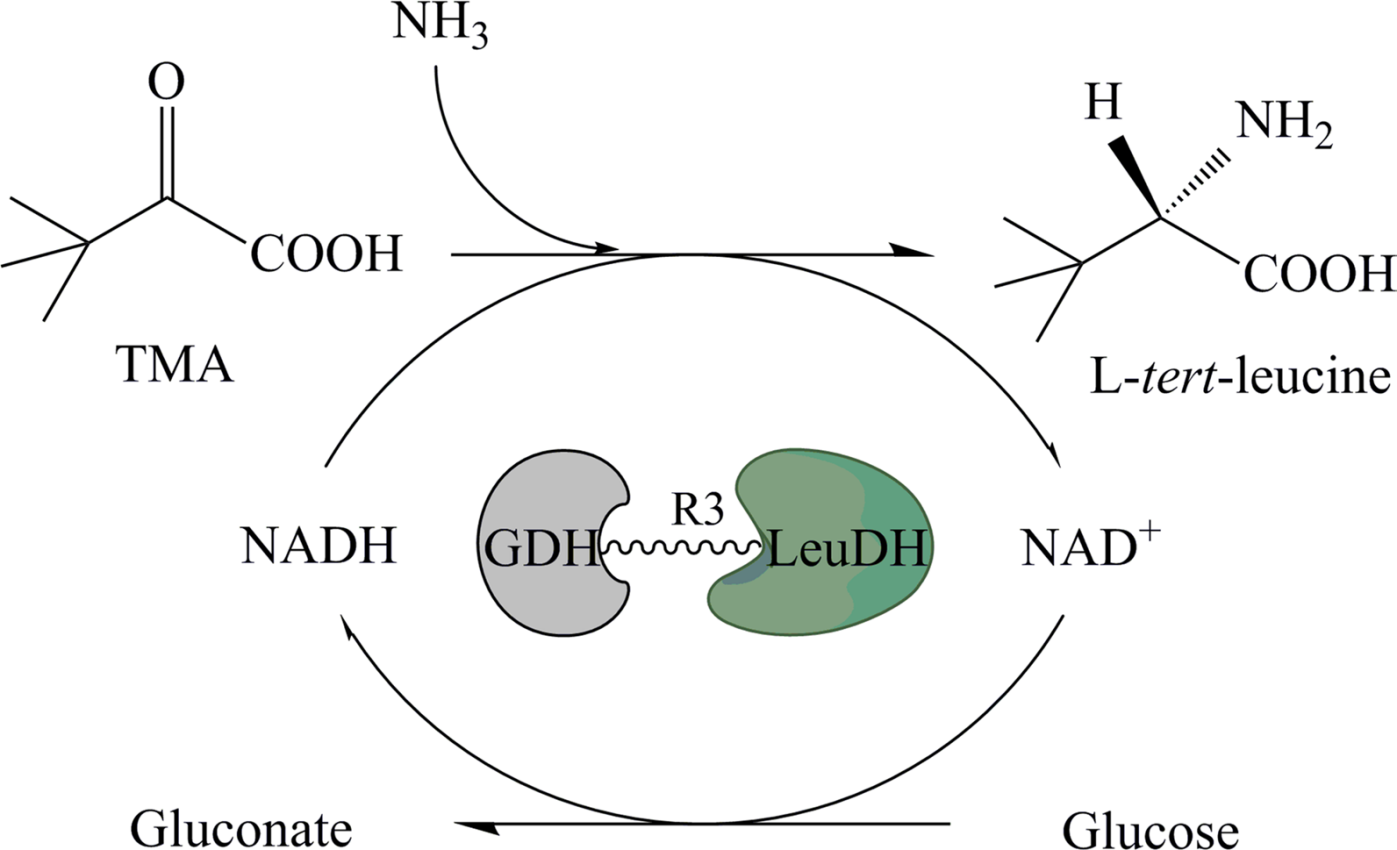
Reductive amination of trimethylpyruvic acid using GDH–LeuDH fusion enzyme for the biosynthesis of optically pure l-*tert*-leucine

There were some bifunctional enzymes can individually catalyze cascade reactions. For example, the chlamydial DapF had function of both racemase and epimerase [23]. Through gene fusion, an engineering bifunctional enzyme could be constructed with two functions and able to catalyze coupling reaction. In this paper, we constructed a bifunctional fusion enzyme, in which LeuDH and GDH played the roles of producing l-tle and cofactor regeneration respectively (Scheme 1). After purification, the properties of novel fusion enzyme, which was expressed in *E. coli* were tested and compared with that of free enzyme. The obtained GDH–R3–LeuDH fusion enzyme had shown improved catalytic efficiency and stability in l-tle production which make it a candidate for further industry utilities.

## Results

### The construction of fusion enzyme and free enzymes

**Fig. 1:**
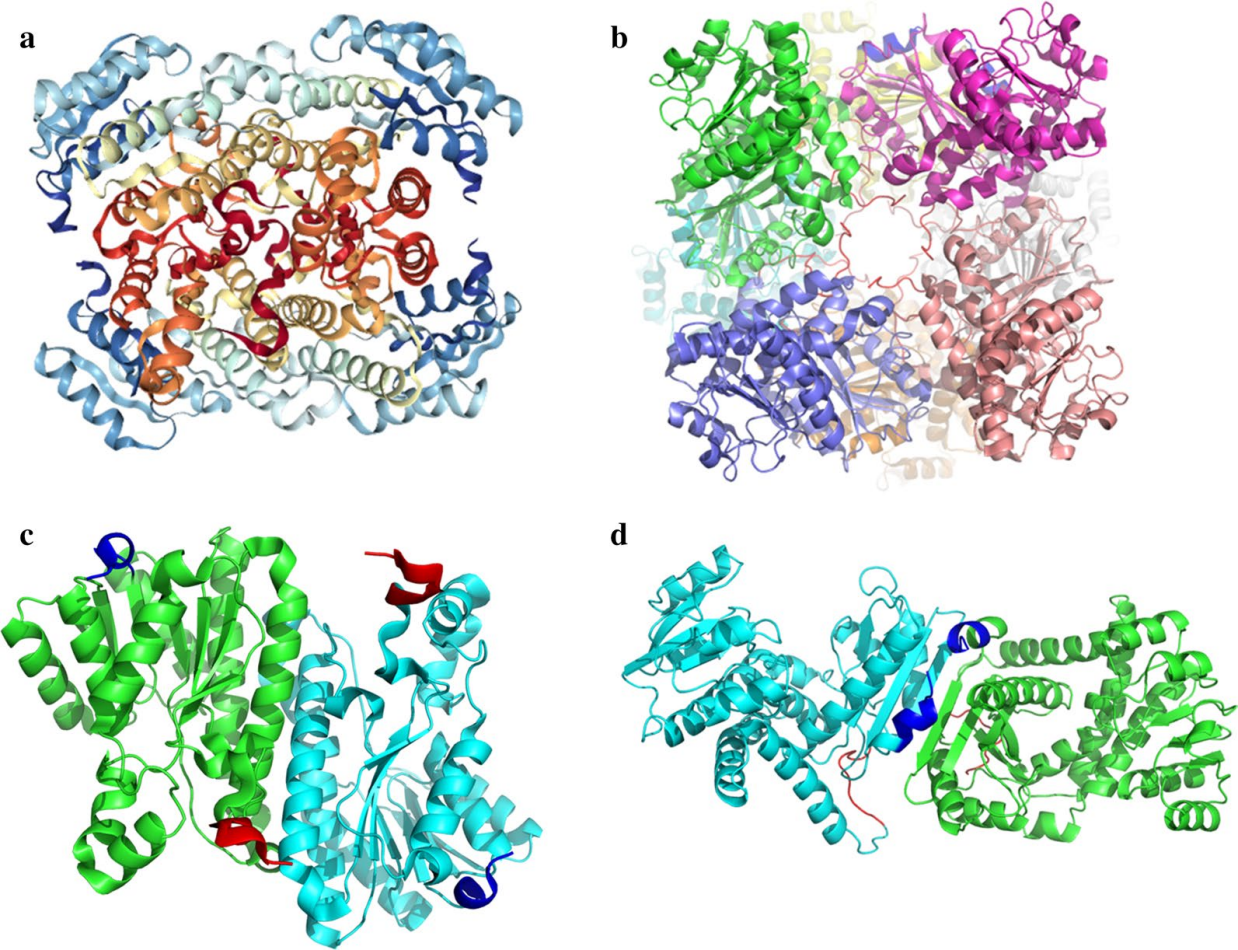
The PDB structures of GDH and LeuDH. **a** GDH from *Bacillus megaterium* (PDB ID: 1GCO, sequence identity: 96.69); **b** LeuDH from *Bacillus sphaericus* (PDB ID: 1LEH, sequence identity: 76.92); **c** the N/C-terminus of GDH; **d** the N/C-terminus of LeuDH; N-terminus (blue), indicates C-terminus (red)

**Fig. 2:**
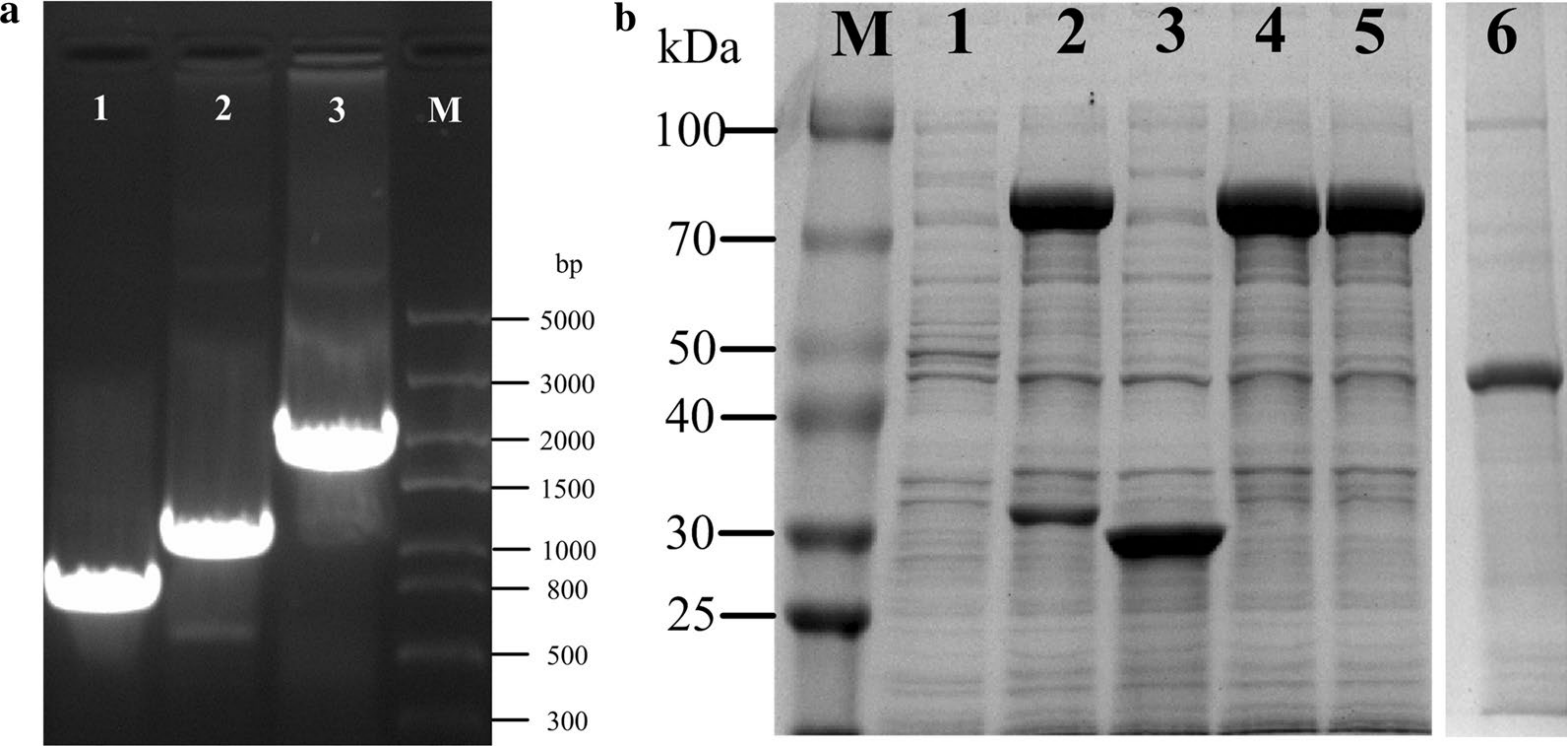
The structure of recombinant *E. coli*. **a** Electrophoresis of PCR products and overlap PCR products of *gdh* and *leudh* (Lane: M, DNA marker; Lanes: 1, *gdh*; 2, *leudh*; 3, *GDH–R3–LeuDH*). **b** SDS-PAGE analysis of cell-free crude extracts of IPTG-induced recombinant *E. coli* (Lane: M, molecular weight marker; lane 1, control group; lane: 2, 4, 5, expression of GDH–R3–LeuDH fusion enzyme; lane: 3, expression of GDH; lane: 6, expression of LeuDH)

The GDH (PDB ID: 1GCO) is a tetramer and the LeuDH (PDB ID: 1LEH) is an octamer in our study (Fig. 1a, b), meaning that the integrity of oligomeric structure is an important factor for maintaining enzyme activity in the process of fusion [24]. Through the oligomeric structures of GDH and LeuDH, we found that the N-terminus of GDH participates in the formation of GDH tetramers, while the C-terminus of LeuDH participates in the formation of LeuDH octamer (Fig. 1c, d). The PDB analysis showed that the fusion sequence from GDH to LeuDH would be benefit for the integrality of both enzyme structures. After determining the order of fusion, the fusion gene of GDH and LeuDH was inserted into a sequence of rigid peptide linker (EAAAK)3 through OE-PCR (Overlap Extension Polymerase Chain Reaction). Finally, the fusion enzyme (GDH–R3–LeuDH), GDH and LeuDH were all expressed in recombinant *E. coli* successfully and obtained the pure enzymes through purification of His-Trap column (Fig. 2).

### The enzymatic properties of fusion enzyme and free enzymes

**Fig. 3:**
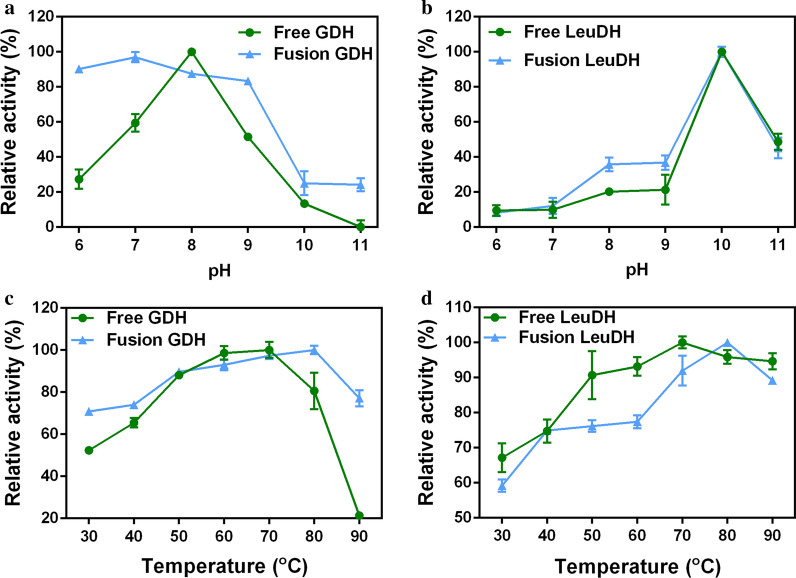
Effects of pH and temperature on enzyme activities. **a** Effects of pH on GDH; **b** Effects of pH on LeuDH; **c** Effects of temperature on GDH; **d** Effects of temperature on LeuDH. Enzyme assays were performed in 0.8 M NH_4_Cl-NH_3_·H_2_O buffers or PBS buffers of different pH at 30 *°*C, using TMA and NADH as substrates (for LeuDH) or glucose and NAD^+^ as substrates (for GDH). Enzyme assays were performed in 0.8 M NH_4_Cl-NH_3_·H_2_O buffers (pH 10.0) or PBS buffers (pH 7.4) of at different temperatures, using TMA and NADH as substrates (for LeuDH) or glucose and NAD^+^ as substrates (for GDH). Free enzyme (circles), fusion enzyme (triangles). Data represent the mean ± standard deviation of triplicate samples. Relative activity was based on a percentage of maximum activity under the experimental conditions

After obtaining the fusion enzyme, comparing the enzymatic properties of the fusion enzyme and the free enzyme could evaluate the effect of the fusion. The activities of fusion enzyme (GDH–R3–LeuDH), GDH and LeuDH were determined in various pH values. Reducing the activity of fusion GDH was observed higher than 80% in a pH range of around 6.0–9.0, while that of free GDH only retained 27.4% at pH 6.0 and 51.5% at pH 9.0 (Fig. 3a). The highest oxidizing activity of fusion and free LeuDH were all observed at pH 10.0, while the activity of fusion and free LeuDH only left 35.8% and 20.2% at pH 8.0, respectively (Fig. 3b). This result indicated that the fusion enzyme had better pH adaptability compared with free enzymes.

The effect of temperature on the GDH–R3–LeuDH, GDH and LeuDH were examined in the temperatures range from 30 to 90 °C. The optimal temperature for GDH and LeuDH of fusion enzyme were both 80 °C, while free GDH and LeuDH were both 70 °C (Fig. 3c, d). Additionally, GDH of fusion enzyme retained more than 70% of the highest activity at the temperatures ranged from 30 to 90 °C, while free GDH only retained 52.4% at 30 °C and 21.4% at 90 °C (Fig. 3c). The results demonstrated that the fusion enzyme might be applied for biosynthesis reactions at a higher temperature.

**Fig. 4:**
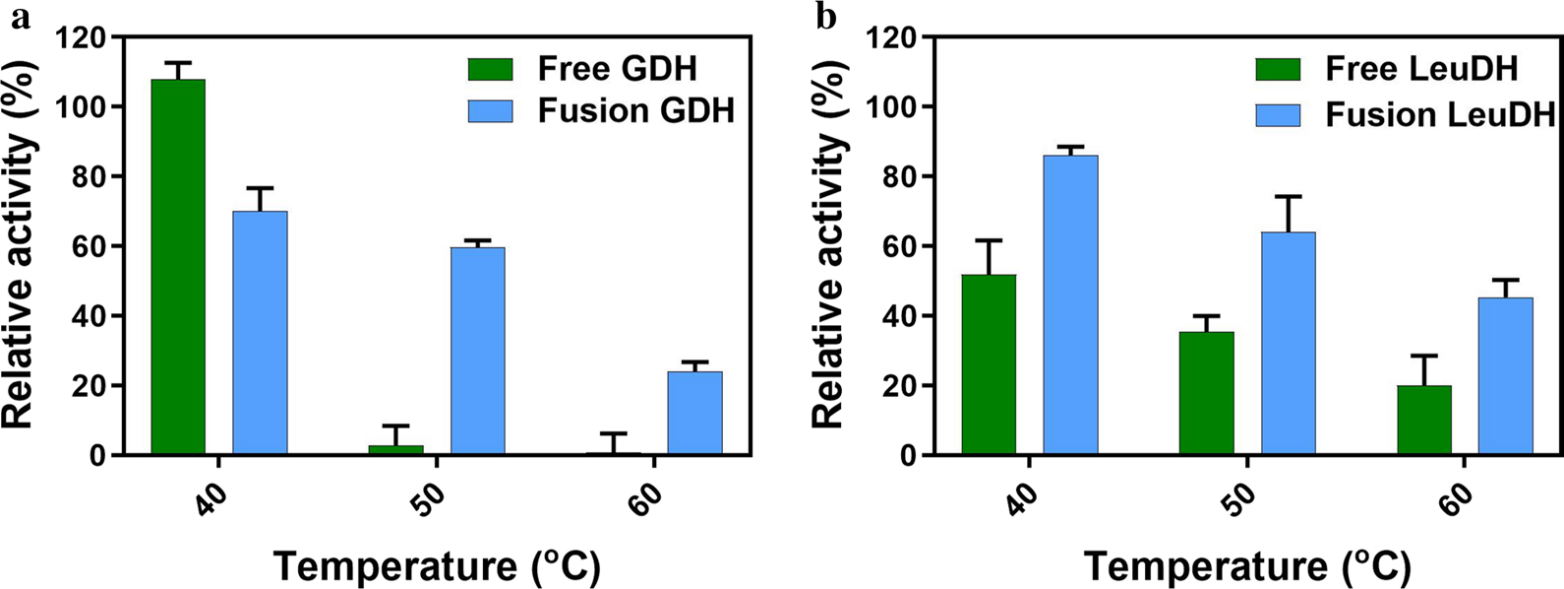
Thermal stability of enzymes. **a** Free GDH and fusion GDH; **b** Free LeuDH and fusion LeuDH. Enzyme assays were performed in 0.8 M NH_4_Cl-NH_3_·H_2_O buffers (pH 10.0) or PBS buffers (pH 7.4) of at different pre-incubated temperatures for 1 h, using TMA and NADH as substrates (for LeuDH) or glucose and NAD^+^ as substrates (for GDH). Free enzyme (circles), fusion enzyme (triangles). Data represent the mean ± standard deviation of triplicate samples. Relative activity was based on enzyme activity before treatment as one hundred percent

The residual activities of the fusion enzyme and free enzymes were determined after incubating at 40, 50 and 60 *°*C for 1 h. As shown in Fig. 4, the residual activity decreased with temperature increasing. Free GDH was almost inactivated at a temperature higher than 50 *°*C, while fusion GDH retained 60% and 20% activity at 50 and 60 *°*C, respectively (Fig. 4a). Fusion LeuDH maintained higher enzyme activity than free LeuDH in different incubating temperatures (Fig. 4b). The fusion enzyme exhibited significant thermal stability both in GDH and LeuDH moieties compared with free GDH and LeuDH, indicating that the fusion enzyme was beneficial for stabilizing fused moieties. The improved environmental adaptability and thermal stability of the fusion enzyme would have a positive impact on the catalytic performance of the coupling system.

### The catalytic performance of fusion enzyme and free enzyme system

**Fig. 5:**
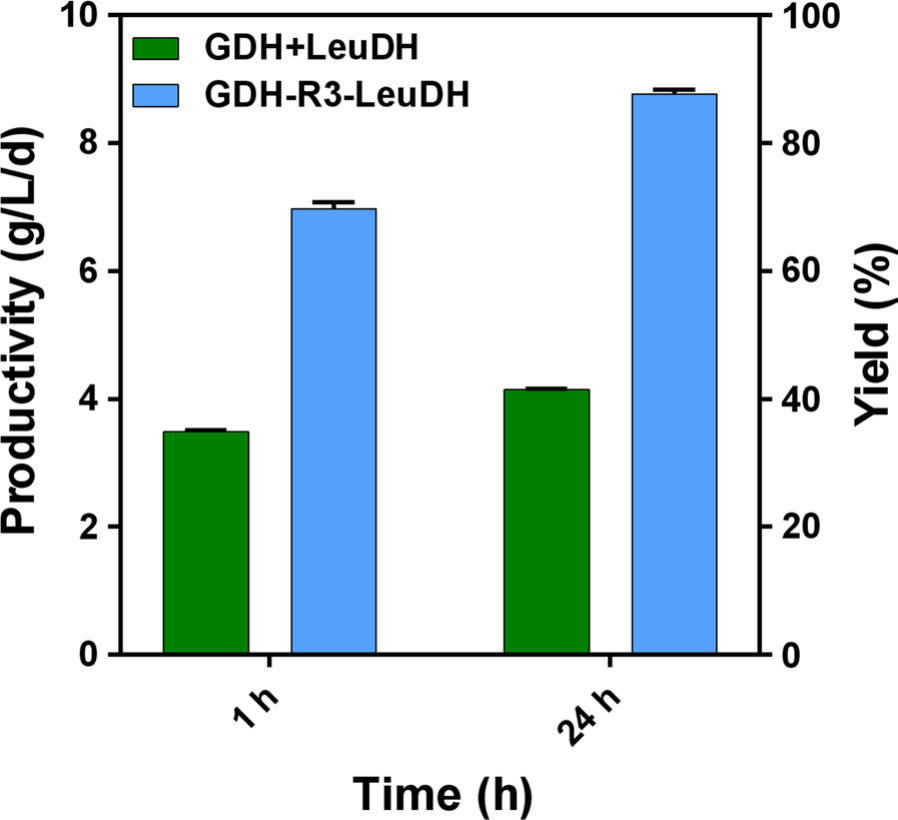
The synthesis of l-tle catalyzed by fusion enzyme or free enzyme system. The enzyme activities were kept as equal for both reaction systems. Reaction conditions: fusion enzyme or free enzyme system, 100 mM substrate, 0.4 mM NAD^+^, 30 *°*C, pH 9.0, 200 rpm. Yield means the ratio of the synthesized l-tle actually to the synthesized l-tle theoretically. Data represent the mean ± standard deviation of duplicate samples. Free enzyme system (green), fusion enzyme (blue)

To study the catalytic performance of different multi-enzyme coupling systems, the synthesis of l-tle was implemented with two sets of biocatalysts, fusion enzyme and free enzyme system. The concentrations of synthesis of l-tle were determined by the standard curve of l-*tert*-leucine by HPLC (Additional file 1: Fig. S1). The productivity of l-tle synthesized by fusion enzyme in 1 h was about 200% as much as that of the free enzyme system, indicating fusion enzyme had a higher initial catalytic rate. After 24 h, the yield of l-tle synthesized by fusion enzyme could achieve about 90% while the free enzyme system was only about 40%, meaning the catalytic efficiency of the fusion enzyme was better than that of the free enzyme system (Fig. 5). The advantages of fusion enzymes were reflected in the catalytic rate and yield compared with free enzyme system.

### The biosynthesis of l-tle by fusion enzyme

**Fig. 6:**
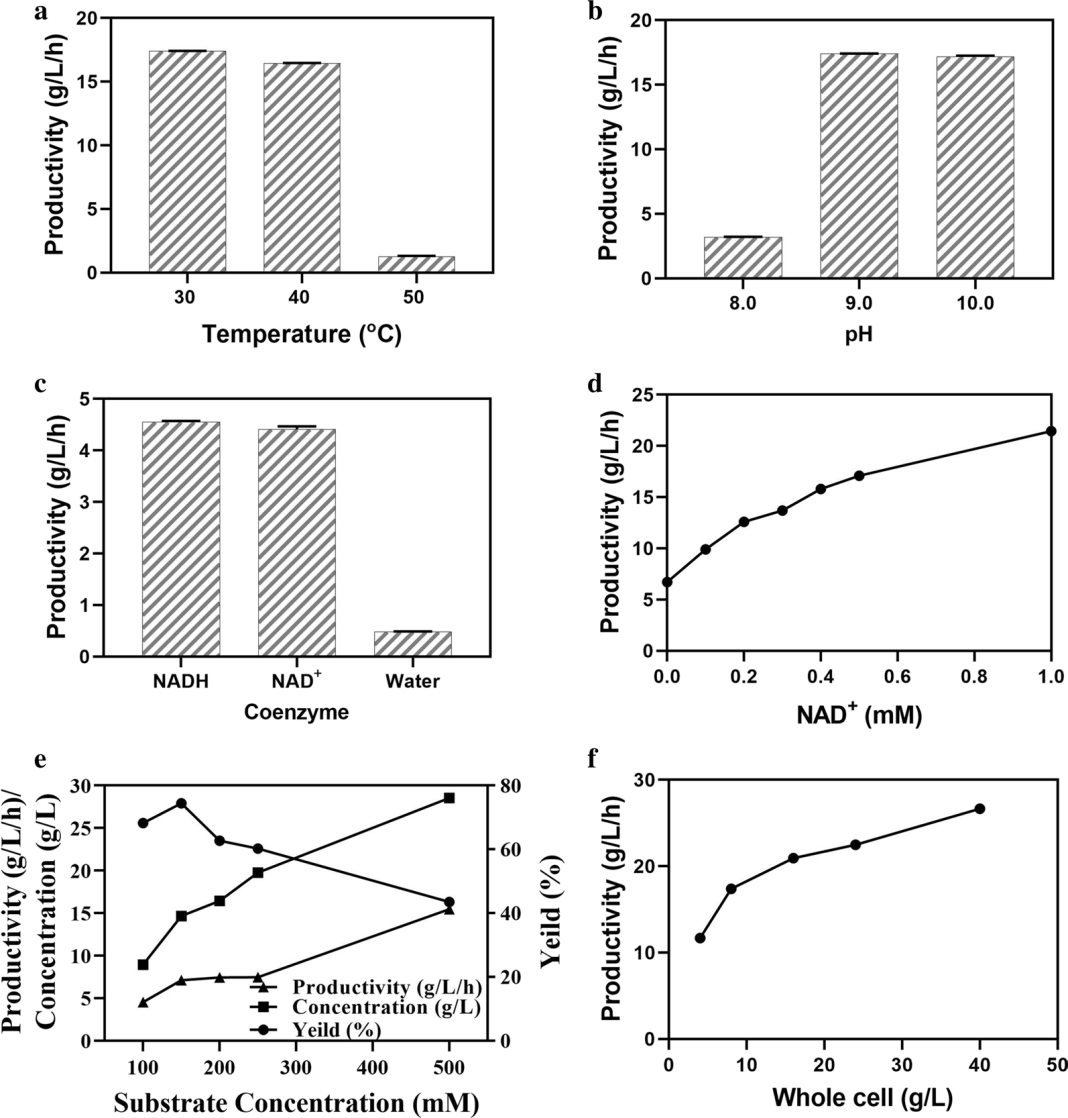
The synthesis of l-tle catalyzed by fusion enzyme whole cells. **a** 2 mL reaction system including 8 g/L whole cells, 500 mM substrate and 0.4 mM NAD^+^ under 30/40/50 *°*C, pH 9.0, 200 rpm. **b** 2 mL reaction system including 8 g/L whole cells, 500 mM substrate and 0.4 mM NAD^+^ under 30 *°*C, pH 8.0/9.0/10.0, 200 rpm. **c** 2 mL reaction system including 8 g/L whole cells, 100 mM substrate and different kinds of cofactor under 30 *°*C, pH 9.0, 200 rpm. **d** 2 mL reaction system including 8 g/L whole cells, 500 mM substrate and different concentrations of NAD^+^ under 30 *°*C, pH 9.0, 200 rpm. **e** 2 mL reaction system including 8 g/L whole cells, different concentrations of substrate and 0.4 mM NAD^+^ under 30 *°*C, pH 9.0, 200 rpm. Productivity (triangles), concentration (squares), yield (circles). (f) 2 mL reaction system including different concentrations of whole cells, 500 mM substrate and 0.4 mM NAD^+^ under 30 *°*C, pH 9.0, 200 rpm. Productivity indicates the concentration of l-tle synthesized in 1 h. Data represent the mean ± standard deviation of duplicate samples

To obtain the optimal reaction conditions for the biosynthesis of l-tle, the effect of reaction conditions on the catalytic efficiency was investigated. Considering the investigation of the optimal pH and temperature and thermal stability of the fusion enzyme, the most suitable catalytic of pH and temperature might be between 8.0–10.0 and 30–50 *°*C, respectively. The whole cells were used to synthesize l-tle in all experiments in this part. The results showed that the catalytic efficiency at pH 9.0 and 10.0 was significantly higher than that at pH 8.0 for the low activity of LeuDH moieties of fusion enzyme at pH 8.0 (Fig. 6a). The reaction rate and yield were extremely low at 50 *°*C (less than 10%) for the whole cells might inactivate rapidly under the reaction condition (Fig. 6b). The productivity of l-tle at 40 *°*C was higher than that at 30 *°*C in a short time (1 h), but the final yield of l-tle was still below 30 *°*C. Finally, pH 9.0 and 30 *°*C were the preferred reaction conditions.

The effects of different kinds and concentrations of cofactor on the catalytic reaction were investigated. Regardless of adding NADH or NAD^+^, the catalytic rate and yield of fusion enzyme were equivalent, while the fusion enzyme showed scarcely any catalytic efficiency without the addition of cofactor (Fig. 6c). With the increase of NAD^+^ concentration, the productivity increased linearly firstly and became equilibrating after reaching 0.4 mM, which indicating that the concentration of NAD^+^ was saturated (Fig. 6d).

**Fig. 7:**
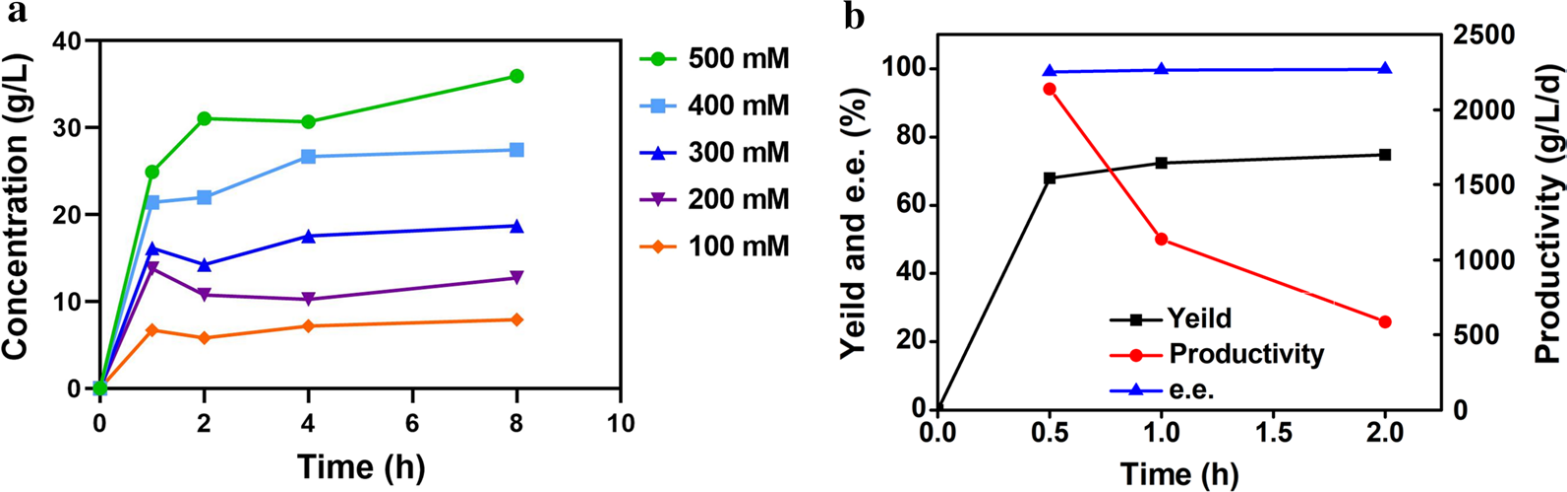
The synthesis of l-tle catalyzed by high content of fusion enzyme whole cells. **a** 2 mL reaction system including 40 g/L whole cells, different concentrations of substrate and 0.4 mM NAD^+^ under 30 *°*C, pH 9.0, 200 rpm. 500 mM (green), 400 mM (wathet), 300 mM (blue), 200 mM (purple), 100 mM (orange). **b** 200 mL reaction system including 40 g/L whole cells, 500 mM substrate and 0.4 mM NAD^+^ under 30 *°*C, pH 9.0, 200 rpm. Yield (squares), productivity (circles), e.e. (triangles). Data represent the mean ± standard deviation of duplicate samples

Judging from the synthesis of l-tle, the substrate concentration promoted the initial catalytic rate and concentration of l-tle synthesized by fusion enzyme, while the yield of l-tle decreased for the limit of the substrate (Fig. 6 e, Additional file 1: Fig. S2). The productivity of l-tle increased with the increase of the concentration of fusion enzyme, which indicating that the increase in the concentration of fusion enzyme could improve the catalytic efficiency (Fig. 6f). Finally, the substrate catalyzed by fusion enzyme with high activities finished in 1 h at the low concentration and in 4 h at the higher concentration, showing the high content of whole cells had a better catalytic rate (Fig. 7a).

Taking comprehensive consideration of reaction conditions and the effect of substrate concentration, to achieve the application of synthesis of l-tle catalyzed by fusion enzyme, the catalytic reaction was scaled up with the high content of whole cells under a single batch of catalytic experiments. A 200 mL scale system containing 40 g/L of whole cells, 500 mM substrate and 0.4 mM NAD^+^ under 30 °C, 200 rpm and pH 9.0 was built. The yield of l-tle was 67.9% after half an hour of reaction while reached the highest value (74.7%) in 2 h, indicating the synthesis reaction of l-tle catalyzed by fusion enzyme basically completed after half an hour (Fig. 7b). The concentration and space–time yield of l-tle achieved 44.5 g/L and 2136 g/L/day respectively. All the e.e. values of l-tle were more than 99%.

## Discussion

In order to ensure that the synthetic process catalyzed by oxidoreductase who need the expensive cofactor NAD(H) is economically feasible, an efficient cofactor regeneration system is the choice. The cofactor regeneration systems are usually constructed by two catalytic entities, and the multi-enzyme complex could shorten the spatial distance of catalytic entities to accelerate the regeneration of cofactors [25, 26]. Fusion enzyme is an effective method to realize the construction of multi-enzyme complex, while how to maintain the enzyme activities of two enzyme is the most difficult problem. Firstly, by analyzing the PDB structure, we determined the sequence fusion sequence from GDH to LeuDH, so that the integrity of the oligomerization interface of each oligomerase could be maintained. Then, in order to prevent the two enzymes from interacting with each other during the folding process, it is essential to add a peptide link to mediate [18, 27].

The peptide linkers, mainly includes flexible peptide and rigid peptide, were used to build fusion proteins in many studies. A fusion protein MBP-(EAAAK)3-GFP-(GGGGS)3-HepA was constructed by (GGGGS)3 and (EAAAK)3 to obtain a complex with good activity and thermal stability [28]. In order to maintain the two molecules in a reasonable distance, a (EAAAK)3 was used to fuse the β-glucanase (Glu) and xylanase (Xyl) double enzymes and got a Glu-xyl fusion enzyme including the dual enzyme activities [27]. The sequence (EAAAK)3 could form α helix and provide a rigid peptide linker to keep the oligomers of fusion enzyme in the proper distance with good behavior and improve the structural stability. Therefore, the fusion enzyme fused the sequence from GDH to LeuDH mediated by (EAAAK)3. Finally, the activities of GDH and LeuDH in fusion enzyme were 0.80 U/mg and 0.99 U/mg respectively. The results indicated that fusion enzyme mediated by a rigid linker with the right fusion sequence could keep the enzyme activities and reduce the difference of activities between GDH and LeuDH to enhance the efficiency of the coupling system.

For the enzyme-catalyzed reaction process, maintaining the stability of the enzyme activities is essential for the catalytic efficiency. Fusion enzyme could make the protein structure of multi-enzyme more stable performancing in the adaptability of enzymes including pH and temperature [29], which exhibited significant enzyme activities in a wide range of pH and temperature and excellent thermal stability in our study (Figs. 3, 4). It had been reported that the fusion of two multimeric enzymes could lead to the formation of a multimeric protein network [30] and the engineered bifunctional fusion proteins compose of two multimeric enzymes could form oligomers [31]. In this work, LeuDH and GDH usually form in octamer and tetramer respectively, then the implementation of GDH–R3–LeuDH could form a multimeric state which explained the enhance of stability of the fusion enzyme. It meant the catalytic reaction of GDH–R3–LeuDH could maintain last longer and reduce the regulation of the reaction system profit from the improvement of environmental tolerance and thermal stability.

The fusion structure not only gave the multi-enzyme complex excellent stability, but also shortened the spatial distance between the two enzymes. As we all known, the substrate channeling effect was a mechanism about intermediates delivery in multi-enzyme complexes [32], and it allowed the direct transfer of intermediate from one enzyme to an adjacent enzyme and decreased the diffusion of intermediate into solution [18, 33]. The productivity of l-tle synthesized by GDH–R3–LeuDH was twofold of free enzyme system, which indicated the acceleration of fusion enzyme in the catalytic reaction of l-tle synthesis (Fig. 5). The fusion structure bridged the double-enzyme to form substrate channeling effect and make the transfer of cofactor quicker. Compared with free enzyme system, the higher final yield of l-tle synthesized by GDH–R3–LeuDH illustrated the fusion enzyme could maintain the operation of coupling system and enhance the efficiency of the l-tle synthesis.

To establish the catalytic system of GDH–R3–LeuDH whole cells, the effects of reaction conditions, cofactor, and the concentration of substrate on l-tle synthesized by whole cells were investigated separately (Fig. 6). The optimum conditions for the catalytic system were: pH 9.0, 30 *°*C, 0.4 mM (NAD^+^) and 500 mM (substrate). Although the structure of GDH–R3–LeuDH was stable, the optimum reaction conditions on the catalytic system were still mild and tallylied with natural enzymatic reaction [34]. The great environmental tolerance and thermal stability indicated that the fusion enzyme system could realize the synthesis of l-tle without controlling the temperature and pH in the catalytic process. The low concentration of NAD^+^ could maintain the catalytic reaction of the coupled system, indicating the NADH regeneration by GDH suited for the catalytic efficiency of LeuDH and reduced the cost of cofactor. The results showed that the increasing of substrate concentration would influence the initial catalytic rate and yield of l-tle synthesized by GDH–R3–LeuDH (Fig. 6e), for the substrate concentration is an important parameter in enzymatic reactions and it could influence the full utilization of enzyme activity or result in substrate inhibition [4, 35].

The substrate inhibition has always been a major problem that plagued the scale-up in industries [35]. In our study, the productivity of l-tle increased with increasing the content of whole cells (Fig. 6 f), meaning that increasing the activities of GDH–R3–LeuDH may be a useful strategy to weaken the substrate inhibition. After using the high content of whole cells (40 g/L) to catalyze high concentration substrate, the l-tle was synthesized in a short time (Fig. 7 a), which indicating the strategy would increase the catalytic rate of l-tle in the high concentration substrate and reduce reaction time to save costs. So, in order to scale up the catalytic reaction better, the high content of whole cells (40 g/L) were used as the catalyst concentration to realize the maximization of l-tle production efficiency.

According to the report on the biosynthesis of l-tle by multi-enzyme coupling system, a LeuDH and FDH free coupling system established by Degussa AG achieved the l-tle space–time yield of 638 g/L/day [14], while the LeuDH-FDH enzyme complex (RSLF) could only obtain l-tle with the space–time yield of 57 g/L/day in 24 h [36]. A BcLeuDH/FDH whole cell-catalyst was used to achieve the space–time yield of 109 g/L/day [12] and the co-expressed EsLeuDH and BmGDH whole cells could obtain l-tle with the space–time yield of 945 g/L/day in 1.3 h [13]. Compared with those reported cofactor regeneration systems, whether the enzyme coupling system or whole-cell system, this work could achieve the l-tle space–time yield of 2136 g/L/day in a short time which is the highest reported currently (Fig. 7b). The catalytic efficiency of l-tle by fusion enzyme whole cells is conducive to optimize catalytic reaction and industrialization.

## Conclusions

In our study, we applied a rigid peptide linker to construct a bifunctional fusion enzyme containing GDH and LeuDH moieties and compared the properties of the fusion enzyme with that of the free enzymes, including structural properties, enzyme activities, thermal stability and ability of synthesis of l-tle. The new fusion enzyme system (GDH–R3–LeuDH) can stabilize the structure of the dual enzyme, improve the adaptability and stability of the coupling system, keep the dual enzyme close enough to obtain excellent proximity effects, increase the cofactor regeneration efficiency and obtain a good catalytic efficiency of synthesis of l-tle. Finally, the space–time yield of l-tle by GDH–R3–LeuDH whole cells attained 2136 g/L/day which is the highest in current research. The GDH–R3–LeuDH fusion enzyme with cofactor regeneration has the potential application for the enzymatic production of l-tle in food and pharmaceutical industries.

## Materials and methods

### Materials

Primer STAR Max DNA Polymerase, restriction enzymes, ligation enzyme were purchased from Takara (Shiga, Japan). pET-28a (+) expression vector was provided from Novagen. Plasmid, gel extraction, and PCR purification kits were provided from Omega bio-tek(USA). *E. coli* BL21 (DE3) competent cell, Isopropyl-β-d-thiogalactopyranoside (IPTG), kanamycin and SDS-PAGE kit were purchased by Transgen (Beijing, China). Modified Bradford Protein Assay Kit, LB broth, d-glucose, NADH and NAD^+^ were purchased by Sangon biotech (Shanghai, China). Trimethylpyruvic acid, l-tert-leucine and D-tert-leucine were bought from Sigma-Aldrich (USA).

### Gene synthesis

Using *E. coli* as the expression host, the amino acid sequence of LeuDH from *Bacillus cereus* (Sequence ID: WP_000171362.1) was optimized, and then synthesized by Sangon Biotech (Shanghai, China). Using *E. coli* as the expression host, the amino acid sequence of GDH from *Bacillus megaterium* (Sequence ID: WP_033578120.1) was optimized, and then synthesized by Sangon Biotech (Shanghai, China).

### PDB structure analysis

The Swiss Model automated comparative protein modeling server [37] (http://swissmodel.expas). According to the amino acid sequence homology, LeuDH from *Bacillus sphaericus* (PDB ID: 1LEH) and GDH from *Bacillus megaterium* (PDB ID: 1GCO) were regarded as structural templates. The molecular graphics software program PyMOL (http://www.pymol.org/) was used for explaining the structure of the enzyme.

### Gene construction

OE-PCR (Overlap Extension Polymerase Chain Reaction) was used to construct a sequential chimeric gene encoding LeuDH and GDH mediated by a rigid peptide linker. The oligonucleotides sequences encoding the corresponding amino acid sequences and rigid peptide linkers were shown in Additional file 1: Table S1. The construction of GDH–R3–LeuDH (R3 indicated three units of rigid peptide linker, (EAAAK)3) chimeric gene was taken the cloning process. First, the GDH gene from pUC-GDH was amplified using Fusion-P1 (5′-GGAATTCCATATGTACAAAGATCTGGAAGGTAAAGTTGT-3′) with NdeI restriction site (underlined) and Fusion-P2 (5′-GATTTCCAATGTCATTTTAGCAGCAGCTTCTTTAGCAGCAGCTTCTTTAGCAGCAGCTTCGCCACGGCCCGCCTG-3′) with sequences that encoding one repeat of rigid peptide linker (R3, underlined). Second, the LeuDH gene from pUC-LeuDH was amplified using Fusion-P3 (5′-ATGACATTGGAAATCTTCGAATAT-3′) and Fusion-P4 (CCGCTCGAGTTACCGGCGACTAATGATGT) with XhoI restriction site (underlined). Finally, PCR products from the first two amplifications were extracted and used as templates for OE-PCR to obtained GDH–R3–LeuDH chimeric gene using Fusion-P1 and Fusion-P4. Then the obtained chimeric gene and pET-28a (+) were digested with NdeI and XhoI and ligated together. The constructed plasmid was named pET-28a-GDH–R3–LeuDH and then transformed into *E. coli* BL21(DE3).

The gene of GDH and LeuDH was also performed PCR by corresponding primers. the GDH gene from pUC–GDH was amplified using GDH-P1 (5′-GGAATTCCATATGTACAAAGATCTGGAAGGTAAAGTTGT-3′) with NdeI restriction site (underlined) and GDH-P2 (5′-CCGCTCGAGTTAGCCACGGCCCGCCTGGAAGCTC-3′) with XhoI restriction site (underlined). the LeuDH gene from pUC-LeuDH was amplified using LeuDH-P1 (5′-GGAATTCCATATGACATTGGAAATCTTCGAATATCTG-3′) with NdeI restriction site (underlined) and LeuDH-P2 (5′-CCGCTCGAGTTACCGGCGACTAATGATGT-3′) with XhoI restriction site (underlined). The obtained gene and pET-28a (+) were digested with NdeI and XhoI and ligated together. The constructed plasmids were named pET-28a-GDH and pET-28a-LeuDH and then transformed into *E. coli* BL21 (DE3).

### Enzyme preparation and purification

200 mL of LB medium and 40 mg/L kanamycin were added in 1 L shake flask. The transformed *E. coli* BL21 (DE3) harboring fusion enzyme or free enzyme plasmids were cultured at 37 °C, 200 rpm in 1 L shaking flask. The expression of recombinant proteins was induced by the addition of 0.2 mM isopropyl-*β*-D-thiogalactopyranoside (IPTG) when the optical density (OD600) was reached at 0.6–0.8 and the *E. coli* was grown at 25 °C for 16 h. Cell was collected by centrifugation and then resuspended with PBS buffer. Cell was disrupted by ultrasonication and removed by centrifugation at 10,000 rpm, 4 °C for 20 min. Obtained crude cell extract was then added to His-Trap column (His-Trap HP 5 mL, GE Healthcare Corp., Piscataway, NJ, USA) which pre-equilibrated with binding buffer (20 mM sodium phosphate, 0.5 M NaCl, 20 mM imidazole, pH 7.4). The binding buffer was equilibrated the column and eluting buffer (20 mM sodium phosphate, 0.5 M NaCl, 0.5 M imidazole, pH 7.4) eluted at a gradient concentration. Target proteins were desalted and concentrated by Macrosep Advance Centrifugal Devices (cut-off 10 kDa, Pall, East Hills, NY, USA). The purity of the obtained enzymes was tested by 10% (w/v) SDS-PAGE. All the protein concentrations were determined using a modified Bradford Protein Assay Kit (Sangon Biotech Co. Ltd) with bovine serum albumin as standard.

### Enzyme characterization

The Tecan M200 Pro plate reader (Tecan Group Ltd., Männedorf, Switzerland) was used to assay the activity of fusion enzyme, LeuDH and GDH by monitoring the NADH (ε = 6.22/mM/cm) concentrations at 340 nm at 30 °C. For the LeuDH part of fusion enzyme and LeuDH, the assay mixture contained 4.5 mM trimethylpyruvic acid, 0.2 mM NADH, 0.8 M NH_4_Cl-NH_3_·H_2_O buffer (pH 10.0) and a certain amount of enzyme solution. For the GDH part of fusion enzyme and GDH, the assay mixture contained 20 mM d-glucose, 2 mM NAD^+^, PBS buffer (pH 7.4) and a certain amount of enzyme solution. The volume of the reaction mixture was 200 μL in all cases. Reactions were initiated by the addition of NADH or NAD^+^. Enzyme activities were tested in triplicates. One unit of LeuDH and GDH activity was respectively defined as the amount of enzyme catalyzing the consumption or generation of 1 μmol NADH per minute under standard measurement conditions. Protein concentration was assayed using the Modified Bradford Protein Assay Kit.

To study the effect of pH on fusion enzyme and free enzymes, enzyme activities were measured in buffers with different pH (0.2 M potassium phosphate buffer including NH_4_^+^, pH 6–8; 0.2 M NH_4_Cl-NH_3_·H_2_O buffer, pH 8–11). The effect of temperature on fusion enzyme and free enzymes were tested over the temperature range of 30–90 °C. The activity was expressed as relative forms (%) with the maximal value of enzyme activity at a certain pH or temperature as 100%.

### Determination of thermal stability

To determine the thermal stability of fusion enzyme and free enzymes, the purified fusion enzyme, GDH and LeuDH were pre-incubated at 40, 50 and 60 *°*C water bath for 1 h and assayed for the residual GDH and LeuDH activity. The activity which was measured was calculated as a percentage of the original activities assayed before incubation. All measurements were performed in triplicate.

### Biocatalysis of l-tle and product analysis

The standard reaction mixture contained 8 g/L whole cells, 100 mM trimethylpyruvic acid, 100 mM d-glucose, 0.4 mM NAD^+^, pH 9.0 (adjusted by adding ammonia), followed by adding free enzyme mixture or fusion enzyme in a total reaction volume of 1 mL. To study the effect of different conditions on the catalytic efficiency of fusion enzyme, different kinds and concentrations of cofactor and different concentrations of substrate and fusion enzyme were all examined. The reaction was performed at 30 °C with 200 rpm of horizontal shaking if not specifically noted. Aliquots of the reaction mixture were taken and stored in − 80 °C for further analysis.

In order to obtain the concentration of l-tle after a reaction, HPLC was used to analyze the samples. The reaction samples were heat-denatured at 90 *°*C for 10 min, firstly. Then precipitate protein was removed by centrifuging at 12,000×*g* for 10 min. Finally, the supernatant samples were filtered using 0.22 μm filter. The concentration and the optical purity (e.e.  %) of l-tle were determined at 35 °C with HPLC column (Phenomenex Chirex 3126Dpenicillamine). The mobile phase was 2 mM Copper (II) sulfate in water/isopropanol (95:5) at a flow rate of 0.8 mL/min.

## Supplementary Information

**Additional file 1.** Figures and Table S1: The list of oligonucleotide primers.

## Data Availability

The datasets supporting the conclusions of this article are included within the article.

## Abbreviations

GDH: Glucose dehydrogenase
LeuDH: Leucine dehydrogenase
TMA: Trimethylpyruvic acid
l-tle: l-*tert*-leucine
