# Supplementary material for: Construction and characterization of a novel glucose dehydrogenase-leucine dehydrogenase fusion enzyme for the biosynthesis of l-tert-leucine
Source: Microb Cell Fact. 2021 Jan 6;20:3. doi: 10.1186/s12934-020-01501-2 (PMC7788806; doi:10.1186/s12934-020-01501-2)
Supplement: Supplementary file 1 — Additional file 1. Figures and Table S1: The list of oligonucleotide primers. [file 12934_2020_1501_MOESM1_ESM.doc]

Construction and characterization of a novel glucose dehydrogenase-leucine dehydrogenase bifunctional enzyme for the biosynthesis of L-*tert*-leucine

Langxing Liao^1†^, Yonghui Zhang^1, 2†^, Yali Wang^1^, Yousi Fu^1^, Aihui Zhang^1^, Ruodian Qiu^1^, Shuhao Yang^1^, Baishan Fang^1, 3*^

1. Department of Chemical and Biochemical Engineering, College of Chemistry and Chemical Engineering, Xiamen University, Xiamen, P. R. China

2. College of Food and Biological Engineering, Jimei University, Xiamen, P. R. China

3. The Key Lab for Synthetic Biotechnology of Xiamen City, Xiamen University, Xiamen, Fujian, P. R. China

^*^Corresponding author: Baishan Fang

E-mail: fbs@xmu.edu.cn

† These authors contributed equally to this paper.

**Table S1**

| Portein/Peptide | Amino acid sequences (N-C) |
| --- | --- |
| R3 | EAAAKEAAAKEAAAK |
| GDH | MYKDLEGKVVVITGSSTGLGKSMAIRFATEKAKVVVNYRSKEDEANSVLEEIKKVGGEAIAVKGDVTVESDVINLVQSAIKEFGKLDVMINNAGLENPVSSHEMSLSDWNKVIDTNLTGAFLGSREAIKYFVENDIKGTVINMSSVHEKIPWPLFVHYAASKGGMKLMTETLALEYAPKGIRVNNIGPGAINTPINAEKFADPEQRADVESMIPMGYIGEPEEIAAVAAWLASSEASYVTGITLFADGGMTQYPSFQAGRG |
| LeuDH | MTLEIFEYLEKYDYEQVVFCQDKESGLKAIIAIHDTTLGPALGGTRMWTYDSEEAAIEDALRLAKGMTYKNAAAGLNLGGAKTVIIGDPRKDKSEAMFRALGRYIQGLNGRYITAEDVGTTVDDMDIIHEETDFVTGISPSFGSSGNPSPVTAYGVYRGMKAAAKEAFGTDNLEGKVIAVQGVGNVAYHLCKHLHAEGAKLIVTDINKEAVQRAVEEFGATAVEPNEIYGVECDIYAPCALGATVNDETIPQLKAKVIAGSANNQLKEDRHGDIIHEMGIVYAPDYVINAGGVINVADELYGYNRERALKRVESIYDTIAKVIEISKRDGIATYVAADRLAEERIASLKNSRSTYLRNGHDIISRR. |


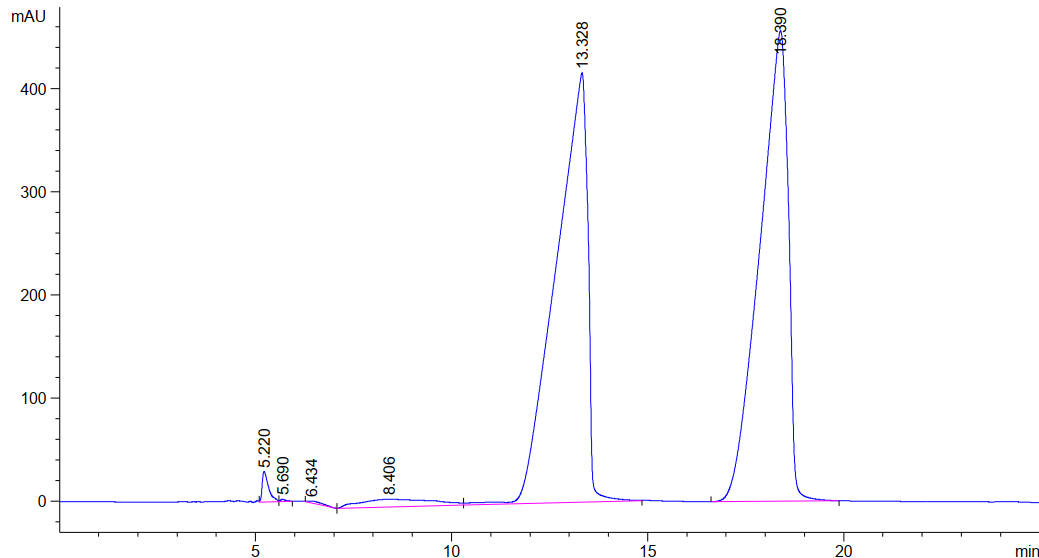


Fig. S1 Analysis of D/L-*tert*-leucine by HPLC

A standard sample of D or L-*tert*-leucine was analyzed by HPLC. The retention time of L-*tert*-leucine was approximately 13 minutes and the retention time of D-*tert*-leucine was approximately 18 minutes


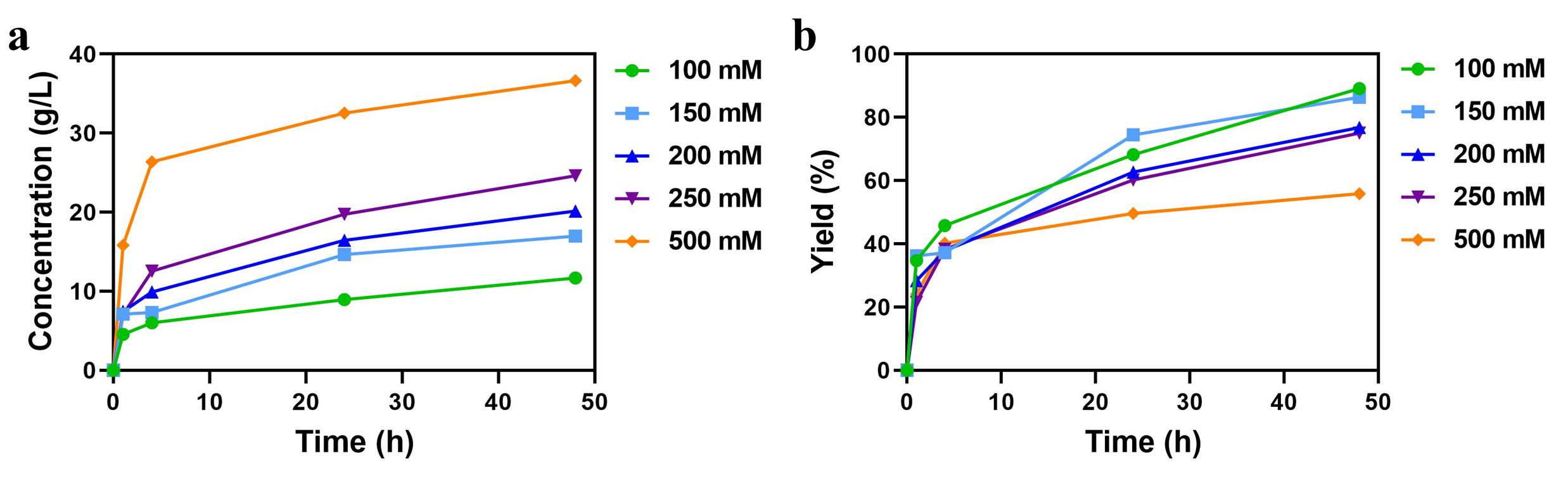


Fig. S2 The synthesis of L-tle catalyzed by fusion enzyme

2 mL reaction system including 8 g/L whole cell, different concentrations of substrate and 0.4 mM NAD^+^ under 30 *°*C, pH 9.0, 200 rpm. (a) the concentration of L-tle. (b) the yeild of L-tle. 100 mM (green), 200 mM (wathet), 300 mM (blue), 400 mM (purple), 500 mM (orange).
